# The politics of irrationality

**DOI:** 10.1111/maq.12809

**Published:** 2023-09-13

**Authors:** Laurie Denyer Willis, Miriam Kayendeke, Clare IR Chandler

**Affiliations:** ^1^ Department of Social Anthropology University of Edinburgh Edinburgh UK; ^2^ Infectious Diseases Research Collaboration Kampala Uganda; ^3^ Department of Global Health and Development London School of Hygiene and Tropical Medicine London UK

## Abstract

In siloed discussions of antimicrobial resistance, antibiotic use on farms in the Global South has emerged as a key site for intervention. The antibiotic consumption targeted is not *all* consumption, but “irrational” consumption. This concept of irrationality is neither new, nor true, but rather is a long‐standing form of maintenance work within global health systems. Via an attention to chickens and the antibiotics farmers use to raise them in the suburbs of Kampala, we suggest that claims of irrationality are a central part of constituting what Tania Li has called the ‘deficient subject’. In other words, irrationality, like the chicken and the antibiotic, is itself a humanitarian device that maintains a certain condition of governance where ‘Africans’ are imagined as being *in deficit* of rationality and good behavior. Claims of irrationality justify (and mask the political nature of) intervention.

## INTRODUCTION

The chickens’ breathing was concerning. They were wheezing, each intake of breath a labor. Helen explains that she called out urgently to her farm worker, Ahram, to come in the middle of the night and listen to these rapid intakes of breath. All her laying hens were experiencing cough, flu, and reduced egg production, and she had found two of them dead the morning before. In the dry season, she says, dry winds and dust blow through the chicken houses causing respiratory issues. A few days later, Helen's son holds to my ear a reddish blonde hen that he's taken from their farm's “sick stack,” saying, “listen, do you hear its distress?” “It sounds phlegmy?,” I (LDW) say, trying to learn to listen. “It's a rattle,” he corrects. “Hold it close to you,” he instructs, shifting the bird away from my ear to my chest. “Don't just listen, feel for the rattle against your body.”

We are on a large chicken farm, located in the suburbs of Kampala, Uganda, inside a three‐story chicken barn with slatted wooden walls. Each floor holds just over one thousand chickens. The structure is located next to an almost identical three‐story barn with another three thousand birds. Helen had woken in the night to sounds she recognized as birds suffering, and quickly, with the help of Ahram, diagnosed them as having a respiratory infection. Helen acted fast, as an untreated respiratory infection can wipe the hens out, rendering both their lives and her investment useless. In the worst‐case scenario, farmers describe losing all their birds – sometimes thousands at a time – and their investment dreams dashed. Their profits decimated. To avoid this, they will sometimes resort to unloading smaller and thus less valuable hens at reduced prices at market, rather than a risk missing a whole investment‐profit cycle. And so they move fast. In the morning, they begin the process of treating the birds with a combination of the broad‐spectrum antibiotics oxytetracycline and enrofloxacin, a technique they've learned from other farmers and perfected over time. Throughout the weekend, the farm workers move through the chicken stacks, supplying the flock with this antibiotic regimen over four more days.

The chicken has played a starring role in global health and development in recent years. Bill Gates, for example, has said that “just about anyone who's living in extreme poverty is better off if they have chickens” (2016), echoing, perhaps accidentally, an American 1928 Republican Presidential campaign slogan of Herbert Hoover, which promised a “chicken in every pot and a car in every garage” (The New York Times, 1928). In both scenarios, the chicken—and its consumption—materially and symbolically signals a shift toward prosperity for all. Indeed, the layer of fossilized chicken bones that today covers the earth is a key marker of how prosperity is made material in the Anthropocene (Bennett et al., 2018). For Gates, and many other global health actors, chickens are one of many magic bullets: they are imagined to act like small humanitarian devices (Collier et al., 2017) that can raise income levels, correct so‐called nutritional deficiencies, and empower women in the Global South.

The modern chicken alone, however—typically raised in captivity and in large quantities—is a finicky bird, prone to illness and death before it has been sufficiently productive in either laying eggs or reaching a size worth eating or selling. The chicken desired in Kampala by consumers and by global health actors like Gates requires a range of inputs. Indeed, as we have documented elsewhere (Chandler, 2019; Kayendeke et al., 2023), scaled‐up chicken farming on small pieces of suburban land in Kampala is typically only successful when entrepreneurs follow what one farmer called “the recipe,” which involved intensively investing in greater numbers of imported breeds, using feed concentrates rather than traditional livestock “foods” (e.g., maize, silverfish, and soya beans), employing rigorous chemical disinfectant processes, and using of a range of antibiotics, both prophylactically and to treat acute infection. And even these inputs only tend to work when deployed alongside the careful labor and attention that an entrepreneur like Helen—and her staff—can give.

But despite the enthusiasm for the chicken, global health actors and funders, from Bill Gates himself (CARB‐X, 2018), to the World Health Organization (WHO) and the United Nations Food and Agricultural Organization, have expressed significant concerns over the use of antibiotics in raising animals throughout sub‐Saharan Africa over the past three decades (WHO, 2015; WHO, FAO, OIE, UNEP 2022). Currently, the amount of antibiotics used in livestock farming globally is understood to exceed the amount used for humans, and there are numerous pathways by which this imbalance can lead to drug‐resistant infections in human populations (Kirchhelle, 2020; Landers et al., 2012). The tensions between antibiotic use for human health and antibiotic use within food production have been discussed for decades, but when it comes to action on antimicrobial resistance (AMR) in sub‐Saharan Africa, the framing tends to focus on a “lack of knowledge” on the part of farmers. Across sub‐Saharan Africa, AMR has been described as an impending crisis, imagined as an “apocalypse” on the horizon of “fragile” healthcare systems (Alvarez‐Uria et al., 2016; Ayukekbong et al., 2017; Collignon et al., 2018; Okeke et al., 2005). Across our ethnographic work in policy meetings, from Kampala, to Bangkok, to London, it was common for us to listen to global health actors from a wide range of institutions, at every level, worry about the use of antibiotics on farms in the Global South, describing them as “hot spots” of antibiotic “misuse”, with farmers described as desperately in need of “behavioral interventions” to correct their “irrationality”. Indeed, what Helen and Ahram did to save their 6000 chickens would be deemed *irrational* because it was both metaphylactic (preventative antibiotic dosing applied to all chickens, rather than selectively dosing only chickens with symptoms) and decided by the farmers themselves, rather than on the instruction of a veterinary professional. Of course, given that there were 6000 chickens housed in tight conditions, selective dosing would likely have been futile, and veterinarian services are both limited and expensive in Kampala. But these structural deficiencies that contribute to antibiotic use were rarely discussed in the policy meetings we sat in, nor was it acknowledged that raising chickens at scale, under conditions that fundamentally require antibiotics to be successful, was a celebrated policy intervention at *different* policy meetings.

Instead, in siloed discussions concerning antimicrobial resistance and global health, antibiotic use has emerged as an area of concern and scrutiny, with both international and national action plans calling for increased monitoring and reduced antibiotic consumption (O'Neill, 2016; WHO, 2015; FAO, 2016). The antibiotic consumption targeted by these interventions is not *all* consumption, but what is often termed “irrational” consumption. This tends to mean something akin to consumption that is marked by uneducated *behavior* (Broom et al., 2020; Chandler, 2019; Denyer Willis & Chandler, 2019; Will, 2018). Under this rubric, small‐holder farms across sub‐Saharan Africa—like Helen's—emerge in global health imaginations as sites of potential risk, where farmers are conceived first and foremost as “uneducated” and “irrational” actors whose “behavior” demands intervention.

This concern over African farmers’ “irrational” antibiotic consumption reveals the colonial frameworks that global health both historically emerges from and still today nurtures in its policies, practices, and deliberations regarding antimicrobial resistance. The claim that African farmers are “irrational” and require “behavior change” in their use of antibiotics does multiple forms of harm, including reinforcing enduring tropes of Africans as subjects in need of management, be it via humanitarian intervention, development initiatives, or colonial governance. In this article, we consider the ways chickens are cared for, and the ways this care comes to be characterized as an irrational site in need of behavioral intervention. This matters because this characterization of irrationality is neither new, nor true. Rather, it is a long‐standing form of maintenance work within global health systems themselves. Claims of irrationality render bodies—both human and non‐human (or, here, farmer and chicken)—subject to intervention. In this article, via an attention to chickens and the antibiotics farmers use to raise them, we suggest that claims of irrationality are a central part of creating what Tania Li (2007) has called the “deficient subject” ‐ the recipient of development's “will to improve”. In other words, irrationality, like the chicken and the antibiotic, is itself a humanitarian device that maintains and preserves a certain condition of governance where certain Africans are imagined as being *in deficit* of rationality and good behavior. This of course mirrors other kinds of deficit imaginaries by which African populations have so often been characterized: nutrition deficits, education deficits, and so on, invoking a gold‐standard comparator located in Western bodies, diets and farms. Here, the charge of irrationality, deficiency, and lack positions populations as subject to surveillance, intervention, and management under an aspirational quest for improvement. The article shows how global health actors redescribe the labor of chicken farmers—which is better explained through a combination of economic logics (development, entrepreneurship, investment) and the more‐than‐economic logic of care work (which involves attention, intimacy, and risk mitigation)—as irrational. This claim of irrationality justifies (and masks the political nature of) of the deficit imaginary that partly organizes global health authorities’ views, policies, and interventions. Throughout this paper, we often turn to the metaphor of “Africans being *charged* with irrationality.” We refer to “African” as a term that in development and health discourses is used to explicitly or implicitly refer to “underdeveloped” populations on the continent, deployed by those with power (whether from outside or inside the continent) to describe those who are subjects of this power. We recognize that for those joining this charge against irrationality, it may not seem like a charge at all, but just common‐sense, or simple logic. Our intention is to draw attention to the heritage of this logic, its embeddedness in the moral legitimacy of calls for education, and the implications for perpetuating the imaginary of the “deficient subject” in the everyday lives of people who find themselves caught in this logic. By recognizing irrationality as a “charge” set in long‐standing precedents, including a history of science that is premised in models of deficiency, we highlight numerous dimensions of development apparatus that have become accepted as unproblematic. Such analysis opens up the possibility for correcting injustice for those wrongly charged as irrational, with the potential for averting further inequities in the response to global health challenges such as AMR.

We move through this paper in careful attention to the ways that irrationality is deployed, as well as to what else exists in these spaces, revealing how kinds of shared nourishment, care, and immunity can emerge and persist in new ways (Langwick, 2018) even under violent and debilitating assumptions of deficit. In the first section of this article, we consider the colonial histories that invent irrationality as an apolitical node that naturalizes intervention, working through post‐colonial scholarship, while also paying attention to how anthropologists have long grappled with the enduring concept of “behavior” in public and global health discourse and practice. What we underscore, then, is the politics of irrationality that permeates discussions of life in the post‐colony. In the second section of the paper, we advance these conversations around irrationality and the post‐colonial to think specifically about the suburbs of Kampala and the national project Operation Wealth Creation, which encourages entrepreneurs to invest in chicken and pig farming as modes of livelihood creation. We show how this project is also tied up in longstanding concerns about the availability of cheap and plentiful sources of protein in Uganda, which are themselves linked to enduring colonial concerns in Uganda, and across sub‐Saharan Africa, regarding the so‐called “protein deficit”. In the final section of the paper, we put this material in conversation with the forms of care and intimacy that emerge nonetheless amidst the assumption of irrationality through a discussion of knowledge and intimacy in the care of chickens. We do not argue that animal care in Kampala existed in a different way in some distant romantic African past outside of relations of consumption, property, and growth, before the colonial insistence on irrationality. Rather, as Radhika Govindrajan (2015) has noted, animal keepers are entangled in intimate and complex ways with their animals; farm animals have always been both kin and food, linked to both care and conviviality, even in death. We explore, then, how antibiotic use in chickens is part of an intimate and relational encounter with chickens that is about an expanded concept of care, concerned with development, entrepreneurship, investment, and also attention, intimacy, and risk mitigation.

## IRRATIONALITY AND “INDIVIDUAL BEHAVIOR”

In a “research‐to‐practice” workshop in Kampala, we gathered together in a large and airy boardroom with other experts to discuss major findings from a range of international research groups tackling the problem of AMR in Uganda, and East Africa more broadly. The group was largely international in composition, with researchers and practitioners, including physicians, nurses, and pharmacists, from across the United Kingdom, United States and Europe taking turns in reporting their findings to international health actors stationed in Kampala. Across a range of sites and studies being reported that day, the upshot of the findings was remarkably similar: interventions were needed to tackle the so‐called rampant “irrational” use and sale of antibiotics in hospitals, informal drug shops, and within farming communities. Some iteration of the idea that “Education is key to achieving rational antibiotic use” appeared in every PowerPoint under the headings “Learnings,” “Next Steps,” or “Policy Relevance.” Attendees nodded along, and the Chair of the meeting summed it all up in the end by concluding that many Ugandans were behaving irrationally regarding antibiotics and that there was urgent need of corrective intervention.

Throughout the research, each of us has engaged in these research‐to‐practice conversations, finding ourselves repeatedly confronted by the notion that irrationality is at the root of the AMR crisis. The written policy and research record is clear on this, too. Overwhelmingly the concept of irrationality is deployed to explain the behaviors of individual users (IACG, 2019; OIE, 2016; O'Neill, 2016; WHO, 2015), with a particular emphasis on the irrationality of users in low‐ and middle‐income countries (Johansson et al., 2016; Mashalla et al ., 2017; Sulis et al., 2020). As Dixon et al. write, “(ir)rationality” functions “as an epistemic framework” within discourses around AMR and, more broadly, forms part of the “architectural blueprints of global health” (Dixon et al., 2021, 2). Expanding on the way this concept seeps into practice, they explain that (ir)rationality is used to justify “surveillance, which renders antibiotic use legible via aggregate data on individual behavior” and “tends to favor restrictive and corrective interventions including training/education, audit/feedback, formulary restrictions guideline implementation and decision‐support technologies” (Dixon et al., 2021, 2). The counterpoint to irrational use, however, seems to be hard to pin down. On the one hand, development approves of farmers as rational if they commit to a market‐oriented model over traditional farming (Berndt, 2015), while on the other hand, farmers are deemed irrational if they do this at the expense of public health priorities such as AMR. Here, farmers’ antibiotic use is only deemed “rational” if it is surveyed and managed by presumed experts, such as veterinarians and agricultural and/or livestock extension officers, and happening within an imagined modern agribusiness frame. Indeed, rationality seems to be a moving target for “poor African farmers” who themselves seem to be deemed irrational *a priori*.

But what is at stake in framing African farmers as engaged in “irrational” behaviors? The charge of irrationality is grounded in colonial histories of governance, and the corresponding focus on individual behavior justifies and naturalizes intervention. Certainly the trope of irrationality is used globally, where irrationality and “individual behavior” are sticky concepts within contemporary approaches to global health, emerging out of ‐and extending‐ colonial logics of care and extraction (premised on a notion of colonialism itself as a humanitarian project) (Benton et al., 2017; Kelly et al., 2017 Anderson, 2014; Yates‐Doerr & Maes, 2019). As Benton et al. (2017) explain, when thinking about global health within a postcolonial landscape, *postcolonial* signals “a temporal and spatial specter . . . notions of the objective, the authoritative, and the universal have been shaped by a history of (Western) technoscientific intervention that has influenced assumptions about difference, modernity, and the future . . . the postcolonial also signals a set of historical relationships and enduring connections . . . vis‐à‐vis political economy, state‐building and development. It provides a shorthand for the common characterization of these spaces in terms of scarcity, lack, and absence” (2017, 455).

This narrative of “irrational” antibiotic use, when applied to the Global South, is a less‐than‐subtle reformulation and maintenance of longstanding colonial imaginaries of “irrational” and “ill‐behaved” populations in the “tropics” (Anderson, 2014; Vaughn, 1991). As the historian of medicine Warwick Anderson (2014) reminds us, analyses of global health cannot be only concerned with binaries of domination and submission, but with the ways that power unfolds on uneasy and constantly shifting grounds; it rarely “flows” effortlessly. Rather, as Nott and Harris explain, using the concept of stickiness instead of flow to analyze global health work and its architectures of power reveals “a form of friction, where historical practices ‘stick’ to modern materialities” (2020, 44). In this context, thinking about the concept of irrationality as doing deficit‐making work underscores why certain organizing logics are made to persist; or how a concept like irrationality maintains certain routes into intervention: it is a concept that helps things stick. In contemporary claims to intervention and improvement, irrationality is glue. The notion of irrationality seems to cling to certain bodies and lives, overdetermining how they are perceived and acted upon in global health. Indeed, Tania Li (2007), working from Foucault's (2004) notion of governmentality, has outlined how the improvement of the subjects of development is “rendered technical” and devised as “apolitical” through the various ways they are cast as enduringly “deficient.”

This management of the deficient population is often tied up with racialization (see Nemser, 2017; Rose, 2007; Stepan, 1991). Concerns over racial taxonomies that mapped hierarchies of rationality and irrationality were foundational to colonial logics and to colonization in practice. As Zakiyyah Iman Jackson writes in *Becoming Human: Matter and Meaning in an Anti‐Black World*, “the logic of conquest, slavery, and colonialism produced a linear and relational conception of human animality” (2020, 26−7). Whereas Europeans were considered moral/rational/political animals, Enlightenment thinking placed Africans in a racial taxonomy premised in their irrationality. Indeed, as Jackson continues, we can trace these concerns to the 1888 Berlin Conference and the “Scramble for Africa” where the notion of *terra nullius* functioned as an “organizing logic” (Jackson, 2020, 243) in European colonial ambitions. Terra Nullius was premised on Locke's conception of those to whom God had ordained the world, namely “the industrious and rational.” The logic of terra nullius aided in authorizing colonialism because within this logic, “indigenous peoples were in a so‐called state of nature: in other words, in a state prior to rationality and self‐governance…The clearing away and extermination of peoples in the name of civilization, development, and progress is inseparable from this idea” (Jackson, 2020, 243). As the political historian Barbara Arneil elaborates, “Locke combines a Protestant concern with idleness with a liberal concern with irrationality (since reason is needed to consent to authority) to create a political theory in which only the ‘industrious and rational’ can claim property and therefore be freemen with political power. Each of these ideas (industry and reason) is not only key to Locke's political theory but delineates the idle and irrational, both at home and abroad, from the industrious and rational” (2012, 496).

Given this history, it may seem surprising, then, to see the concept of irrationality so commonly deployed in the contemporary global health vernacular. What is especially pernicious in these contemporary charges of irrationality is that in framing the issue as a question of individual behavior, it turns it into a question of education, thereby justifying intervention in the form of “re‐education” initiatives. As Arneil (2012) explains, this coupling of irrationality and behavior change is not without historical precedent. Colonial logics propelled the construction of labor and farm colonies where the “irrational” and “idle” were housed and re‐educated to “break them free . . . from their bad customs/habits and teaching them, through education and agrarian labor, to become proper citizens” (Arneil, 2012, 491). To reduce antibiotic use to an individual farmer's ignorant, *irrational* behavioral choice, to be corrected via behavior‐change interventions, is an insidious move, but one that is deeply sticky. Indeed, in this journal a quarter‐century ago, Stanley Yoder (1997) analyzed the same set of sticky concepts—behavior and irrationality. Yoder describes how interventionist projects in global health often position behavior as individual and predictive, knowable via cognitive means, hinged on the individual agent, and able to be positioned on a scale of good and bad, so that the supposed bad behavior might be nudged in the “correct” direction through re‐education.

Yoder suggests that these behavior‐change models and techniques are derived from social psychology, which today continues to reinforce lines of inequality elsewhere in the world (Tompson et al., 2021). Indeed, such models fit well – in their inscription of populations with irrational behaviors coupled with authorities’ desire to correct them – with haunted forms of care in the postcolonial landscape, where rationality and irrationality are codified into a “technoscientific intervention” (Benton et al., 2017). Putting irrationality and individual behavior change at the heart of interventions often has the effect of making apolitical what is in fact deeply political, obscuring how inequality shapes environments, landscapes of care, access to infrastructures, and the availability of good health services. More than this, however, the insistence on irrationality as a motivation for behavior‐change interventions is maintenance work: the charge of irrationality works to maintain and preserve long‐standing colonial logics of supremacy, safeguarding the right to intervene.

## RAISING CHICKENS IN KAMPALA: WEALTH & ENTREPRENEURSHIP & THE ‘PROTEIN DEFICIT’

On June 8, 2016, Bill Gates stood in front of a chicken coop that had been constructed on the 68th floor of 4 World Trade Center in Manhattan to announce his plan to donate 100,000 chicks to developing countries as part of his strategy to end extreme poverty. Posing with chickens as part of the promotion of the project, he later tweeted, “If I was in extreme poverty, I'd want to raise chickens.” In Uganda, a related fervor for raising chickens was being cultivated by the Ugandan government itself. Kampala today is a city processing and shifting, and its suburbs—where this research took place—have emerged as a central site for techno‐political experimentation, newly zoned as sites for intensified farming production on small piecemeal plots. As Jacob Doherty (2019) explains, a vivid campaign of transformation was launched a decade ago by the Kampala Capital City Authority, a ruthless program aiming to transform the city's peoples, its animals, and its landscapes of governance and infrastructure. This disputed transition, Doherty outlines, involves ridding the downtown core of cattle, pigs, goats, chickens, and ducks, and extends, as we've found, to constituting the suburbs of Kampala as spaces of industrial agricultural growth through new land zoning policies that designate suburban locations for contained piggery and poultry and cattle raising and processing.

In the suburbs of Kampala, policies encouraging small‐scale pig and chicken farming have led to an influx of new, often relatively inexperienced farmers who are nevertheless determined entrepreneurs. Operating on distinct scales, with distinct visions for the future, investors or entrepreneurs in these scenarios tend to be either middle‐class Ugandans looking to invest their incomes from their secure work, or less well‐off Ugandans, including teachers and informal workers, also looking for opportunities to raise their incomes and improve their livelihoods. Suburban farming initiatives also present opportunities for unemployed youth to be innovators and create their own jobs. Chickens, then, are about investment, improvement, and aspiration for all involved—though in ways both different and similar to how livestock has previously operated a mode of historic and rural investment, food, and kinship in Uganda (Doherty, 2019). This new wave of farmers is contractually disconnected from wholesale buyers but nevertheless still connected through specific mature animal weight requirements, the selling of live young, and the provision of advice and (often perfunctory) livestock rearing training courses. Most of the chicken farmers we met and worked with were relatively new to farming.

Helen, for example, did not have much previous experience with chickens, or with livestock in general. Yet her work raising chickens, far from the actions of an irrational individual, was sophisticated and demanding, motivated by clear economic logics—and, furthermore, structured by a long history of colonial interventions in Uganda. Part of a large group of new farmers we worked with in the Wakiso suburbs of Kampala, Helen had grown up in the city, without space for raising animals. She described herself not as a farmer but rather as an entrepreneur. At the same time as she managed her 6000 birds, she was also setting up a nursery school space next to her compound as another potential revenue stream. Chickens, she had been told by other entrepreneurs in the area, could turn a profit, with the suburbs providing easy access to an emerging and flexible integration model of chicken industrialism. We found ourselves, then, talking with Helen about supply chain management, how to bypass local feed sellers and make your own, for example, by experimenting with various ingredients and processing techniques, and the ongoing problems with contingent labor. Helen's chicken business was profitable, for now, following the new methods that enable higher yields in smaller suburban spaces—stacking high a larger number of imported “exotic” layer breeds, feeding these birds expensive concentrate feeds (manufactured pre‐made mixes), employing rigorous chemical disinfectant processes, and administering antibiotics. In the first instance, these antibiotics are deployed less to boost growth than to treat current or anticipated infection. But farmers also explained that they were observing how using antibiotics seemed to indirectly promote growth and improve production by reducing illness episodes overall.^1^ These new high‐yield farming techniques also require a particular kind of intimate care: attending to the chickens’ breath in order to distinguish the difference between a “rattle” and merely phlegmy breathing, and knowing when a chicken is in distress and how to intervene—to save both the chicken and the investment it represents. Here, the careful use of antibiotics is neither frivolous nor irrational.

These farmers were keeping more than five hundred chickens at a time, encouraged by the ambitious national strategy called Operation Wealth Creation, which supported entrepreneurial farming in Kampala's suburbs at semi‐industrial levels, as part of efforts to reduce national unemployment, combat poverty, and ensure a supply of affordable protein. Following the logics of Operation Wealth Creation and its predecessor initiatives, these entrepreneurs had self‐funded their start‐up farms, oriented by the promise of “harvesting” money (Kayendeke et al., 2023, 10). This entrepreneurial gambit at raising chickens at scale, for people with limited previous experience with livestock or farming more generally, is not happenstance. Rather, it is connected to the enduring myth of the protein deficit, tied to campaigns to improve livelihoods by ensuring food security, reducing hunger, and promoting certain diets and lifestyles deemed healthful. As Sandra Calkins explains, conversations around malnutrition in Uganda draw on a specific conception of health that “reiterates familiar notions of African deficiency”, where “health as growth” becomes a rubric for parsing the possibilities of national “capacity, vitality and development” (Calkins, 2019, 32).

Foremost among these interventions is the production and distribution of affordable protein for all. While malnutrition is a serious issue in parts of Uganda, others have pointed out that Ugandan diets suffer not because of “protein deficiency” but because of inequitable food pathways.

The “protein deficit”, however, has long functioned as a hinge: a discourse that authorizes interventions across multiple fronts in global health and development (Tappan, 2013). While its roots can be located in colonial logics that position Africans as “lacking,” the protein deficit also works as a supposedly apolitical node for parsing both colonial and modern‐day projects of blame and obfuscation over failure to achieve certain targets or goals (Nott, 2021). For historian Jennifer Tappan (2013), for example, the notion of the protein deficit is an extension of the logic of other deficits in nutrition tied to certain kinds of bodies: the poor, the racialized, the non‐male.

In the twentieth century, protein—or its putative lack—became a cause of concern for British colonial officers, both those stationed in British colonies, and in Whitehall, the British Colonial Office in London. This concern emerged not only from the colonial offices, Tappan (2013) notes but also through an earlier iteration of global health that was deeply invested in the project of needing to “save young lives” in Uganda, where many physicians and scientists had witnessed the dramatic death rates related to the disease kwashiorkor, a severe form of protein malnutrition (Tappan, 2013,106). The historian John Nott (2021) explains that attempts to address this crisis involved a project of delineating kwashiorkor as somehow particularly “African,” which involved downplaying or outright ignoring similar disease presentations in Britain itself at the time. It was a project, Nott explains, that could be understood as transferring blame *from* colonial management *to* Africans themselves—making Africans the primary cause of an illness in order to displace attention from undernutrition emerging from poverty in the colonies. This elision shifted blame to supposedly *irrational* behaviors among African parents. Kwashiorkor, then, was constructed as a disease stemming from acute “protein deficiency” across the African continent that was conceived as both “timeless and endemic” (Nott, 2021, 4). In other words, protein deficiency was naturalized—made to be seen as the outcome of “backwardness.” While colonial nutrition science was presented as objective and universal, the diagnosis of kwashiorkor was never an apolitical project. As Nott outlines, it was always at the same time “inextricable from the politics of empire” (2019, 8), a crucial node for the construction of a hierarchy of bodies and civilizations. Here, as has often been observed, nutrition—good and bad—was used to police and enforce boundaries, to characterize certain cultures and individuals as irrational, but also used to diagnose irrationality at the level of the body itself, naturalizing racialized bodies as inferior. The rules and regulations around what colonial actors understood as good and right to eat and cook tell us, similarly, about what was deemed bad, undesirable and/or disgusting. How we define, consume, and navigate food systems and consuming bodies reveals how these processes are always at once both material and symbolic, entangled in complex political ways that demonstrate how power works and unfolds (Gálvez et al., 2020). Under colonial management, the emphasis on protein deficiency partly worked to obscure the “pervasive upturn in undernutrition, food insecurity and famine which accompanied the transition to colonial capitalism” (Nott, 2021, 556). This was replaced with a depiction of kwashiorkor that “flattened both European and African histories of food and health while also contributing to a reductive construction of African alterity” (Nott, 2021, 4). This alterity, Nott explains, was the point. It allowed the storying of kwashiorkor to be tied to colonial projects of betterment and benevolent administration that sought to keep the African populace in their place.

Today, improving production of and access to protein is a national agenda item in Kampala because it is linked to the broader goal shifting from an economy premised on subsistence activities to one based on large‐scale food and agricultural industries. This aspiration in turn is tied to local conceptions of modernity and affluence in Uganda, and the nation's attempt to cultivate a thriving middle class—one modern enough to consume and produce meat beyond subsistence (Doherty, 2019). At the same time, the mission to correct the protein deficit seems to appeal to international donors and policy makers, but for different reasons. Chickens have emerged as a somewhat surprising contemporary magic bullet in both global health and global development work. Across multiple scales, chickens are held up as a sustainable model for improved livelihoods, for both the human body and the so‐called fragile economies of the Global South. Chickens, as Bill Gates declares, are imagined and enacted as potential remedies to a range of issues, seemingly uniting microfinance initiatives, women's empowerment advocates, nutrition experts, organizations to end hunger, and those concerned with streamlining new industrializing global commodity markets (Gates 2016; Glass et al., 2017). Yet rosy proclamations of the fantastic potential of chicken farming gloss over how agricultural initiatives like those in Kampala emerge out of colonial projects of governance, redescribing such initiatives in terms of supposedly unobjectionable goals such as correcting protein deficiency and bringing about prosperity.

## THE ‘DARLINGS’ OF THE FARM: ON CARE AND ATTENTION

**FIGURE 1:**
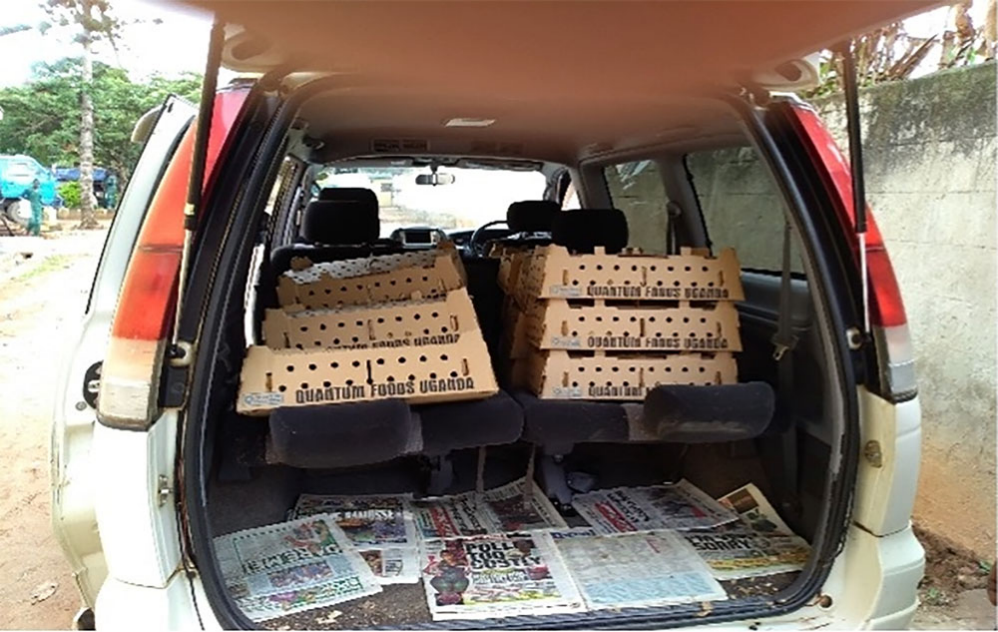
Collecting the day‐old layer chicks. [This figure appears in color in the online issue]

The night has seen steady drizzle, with lightning flashing through the curtains accompanied by bouts of thunder. The drizzle continues into the early hours of the morning. Just as the alarm goes off at 5 a.m., Helen calls to say that she will be late by 30 min, as she has to preheat the brooder. Today we will be collecting one thousand and fifty 1‐day old layer chicks for the farm (see Figure 1).

In the weeks leading up to this point, we have been preparing the brooder itself, an intensive activity that involves converting part of the chicken stacks into a hot room. To do so, Helen had to siphon off a section of the second‐floor stack and clean it with a broad‐spectrum phenolic disinfectant, Virukill, which is sold as a manual cleaning agent for disinfecting chicken storage areas and used as a disinfectant foot bath before entering farm premises or chicken and pig houses. Ahram prepares the disinfectant by mixing it with water in an industrial spray pack, and then loads the heavy equipment onto his back. As he sprays, he makes sure that the liquid disinfectant entirely soaks the surfaces. One spraying can cover about only 1 m^2^, so the task has to be performed 20 times to completely douse the space.

**FIGURE 2:**
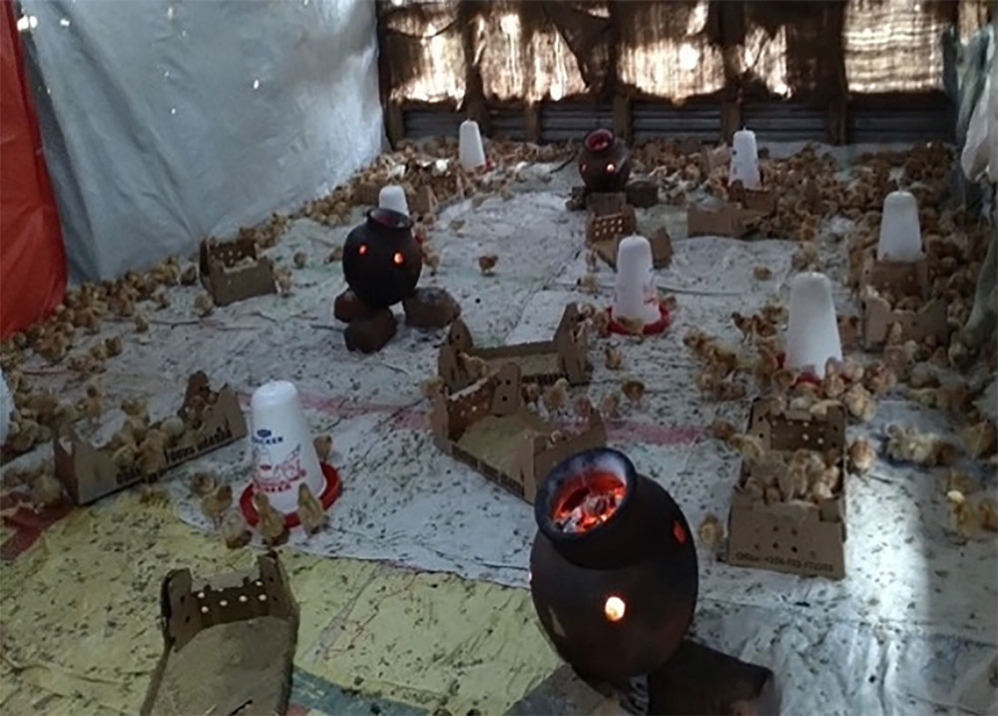
Five locally made pots blazing red hot with charcoal are evenly distributed within the space. [This figure appears in color in the online issue]

Next, we demarcate the large room proposed for the brooder into quarters, using jute and white sacks, and a collection of tarpaulins. First, we nail the jute sacks over the wire mesh to prevent any wind from blowing directly through the brooder, and then we nail the tarpaulins tightly, with no slack, to the wooden poles, enclosing a small space approximately 10 m by 10 m. We then cushion the brooder floor with sawdust and nail clean white polythene sack over the sawdust to create soft bedding. Five locally made pots blazing red hot with charcoal are evenly distributed within the space (see Figure 2). The tarpaulins immediately trap the heat in the room, and our breathing becomes more labored in the enclosed sauna‐like space.

**FIGURE 3, 4 & 5:**
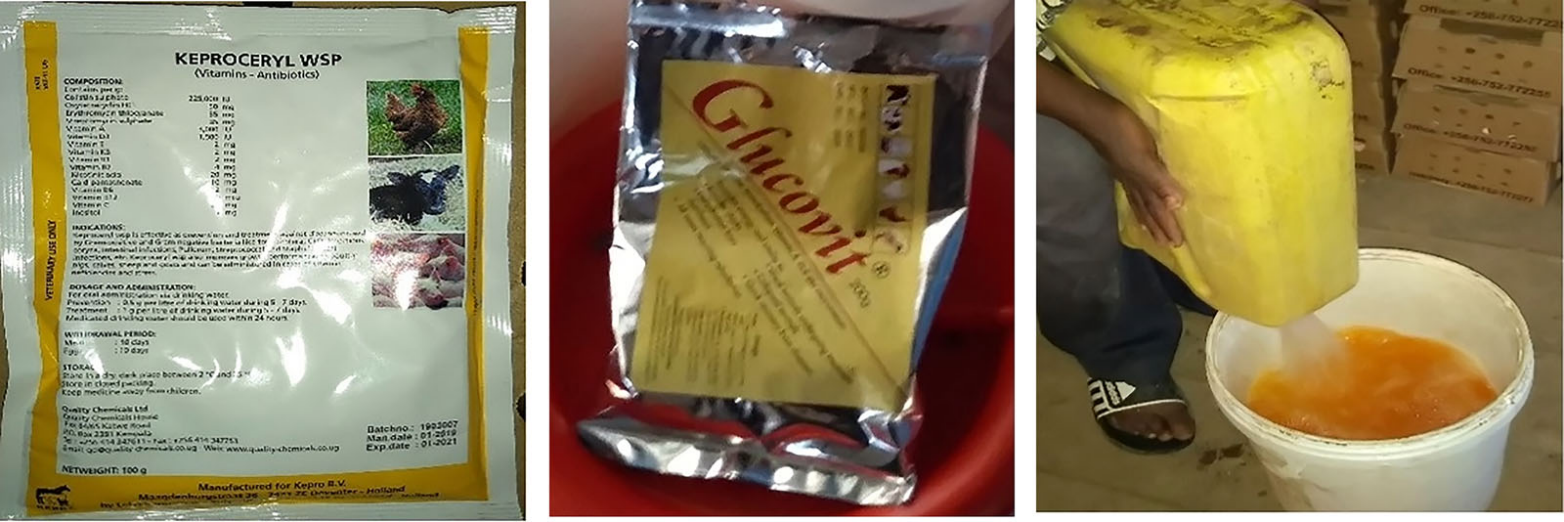
Preparing both Glucovit and Keproceryl powder for the chicks. [This figure appears in color in the online issue]

On our way back to the farm after collecting the chicks, Helen calls on a veterinary officer she knows to come and help out with setting the chicks in the brooder. On arrival, he instructs us to give both Glucovit and Keproceryl powder to the chicks immediately, explaining that the Glucovit raises sugar levels in the chicks and increasing their energy to move around actively. “Chicks come with infections right from the hatchery,” he further explains, and says that a prophylactic dose of Keproceryl will protect the chicks, especially as they acclimatize to this new setting (see Figures 3–5).

The next morning, arriving back at the farm, Ahram and another worker are sleeping right next to the brooder. They have spent the night in the stacks, right next to the new chicks. Their two mattresses are laid down on the floor with bednets rigged up above them. Ahram explains that they will spend their nights here for the month. This helps them to wake up in the night and attend to the chicks without walking a long distance. He explains, too, that being this close to them relieves him of any worry. He is excited to be raising these chicks, he says, and wants to make sure everything goes right. The first week of care is so crucial to their survival that no one wants to take any chances. Before we step into the brooder with Ahram, we notice that the door has been locked and comment on it, never having seen the stacks locked before. Ahram tells me that he decided to lock the door because everyone in the home has been excited about the chickens. Everyone has been making endless trips to the brooder to see for themselves how the chicks are faring, but he wants to ensure that no one can access the brooder without permission. We give them another dose of antibiotics, mixing the five tablespoons of glucose and two tablespoons of Keproceryl powder into a clean bucket and adding it to their 10 L of water.

Back on the farm a few days later—one complete week since the chicks were purchased and placed in the brooder—the new arrivals are still the “darlings” of the poultry farm, as Helen refers to them. Everyone seems to be concerned about how they are faring. Helen is checking on how they spent the night, looking happily at them. She exclaims proudly, “They have grown. They now have wings!”

Remaining with Ahram at the brooder, he explains not only his pride in taking care of the chicks but also why his undivided attention is so critical. Having a defined batch of chickens to care for is a good practice, as the chickens get to know you and you also get to know them. You notice any little thing, are attentive to their sounds, and will spot any small shift in their health fast enough to correct it. He explains that these exotic layers are very sensitive to many things. Even just changing a worker, he says, can cause a drop in the production of eggs, or result in young chicks falling sick. Some chicks might be bullied and scared away at the feeding points, meaning that they will never get enough to eat and will wind up underweight, but if an attentive worker can identify this early and remove any needy chickens to a sick bay where they can be specially fed and monitored for weight gain, potential problems can be avoided.

While these chickens are raised here in the thousands, they are also “darlings” who are doted on. Indeed, Helen and Ahram weave between these rich descriptions of their chickens, urging us to get up close to them and to know them alongside a more actuarial accounting of them. Being attentive to the bodies and lives of chickens, nurturing them, holding them close your own body and taking the time to care for them, seeking out a sick or bullied one, tending to them via sick bays and antibiotics—these are all modes of care that workers like Helen and Ahram engage in, despite the thousands of chickens they labor to maintain. Their care for their chickens is neither novel nor a romantic “African” venture. While chicken farms across Europe, Canada, and the United States, for example, tends to be larger (with tens of thousands of chickens housed together), stories of nurture within these industrial‐commercial operations are not unheard of either. Indeed, Hinchliffe and Ward (2014) detail the ways that farmers actively work with, rather than against, complex microbial environments, in the “making of safe life” for pigs and humans in the United Kingdom. They outline the kinds of “situated knowledge and practices” that animal breeders and farmers deploy to raise healthy pigs, and how this in‐depth knowledge is “obscured and even endangered when biosecurity is reduced to the simple protection of disease‐free livestock” (Hinchliffe & Ward 2014, 136). Very much in common with Helen and Ahram, Hinchliffe and Ward explain that raising and keeping healthy livestock—that is, healthy for humans and for the environments they live in—is a complex dance that is more than just “keeping disease out.” In fact, the relations and interactions of animals, microbes and people are key to *ensuring* health. In Uganda, when policy tries to reduce these complex relations into universal categories called “irrational” or “rational,” it risks being part of the problem, not the solution. How might we instead enable and listen to farmers about how they manage complex and heterogeneous disease threats in their animals that actually manage threats to health—broadly conceived? As Hinchliffe and Ward write, “practitioners are not responsible for biosecurity, but responsive to living complexities and make valuable contributions towards making life safe” (Hinchliffe & Ward 2014, 143). Helen and Ahram, through their care of their darling chickens, were engaged in the work of making life safe, managing threats, and sharing immunity—to the biophysical health of their chickens certainly, but also to the entangled projects of providing care that was attuned to their livelihoods, households, land, and aspirations.

## CONCLUSION

This article has explored the operation of a paradoxical global health project: Ugandans are being encouraged to undertake chicken farming as a means of poverty reduction, while at the same time there is greater investment in national antibiotic surveillance programming, and calls for regulations to prevent the use of antibiotics at a level that would make raising chickens at scale both possible and profitable. This seems like a moment of policy incoherence, or at least bureaucratic fragmentation. On the one hand, raising chickens is being presented as a win for both global heath and global economic development; on the other hand, those same farmers—raising those same chickens—are depicted as potential global health threats, their “irrational” antibiotic behaviors leading to new and resistant infections that could fundamentally alter the course of modern medicine, using in an “apocalyptic” crisis. This tension is evidence of how antibiotics are embedded within particular configurations of social life (Chandler 2019), and how theories of individual behavior change guide approaches to antibiotic use (Denyer Willis & Chandler 2019). But it also demonstrates how the consumption of antibiotics is regulated via imaginaries of deficit. This deficit is articulated via an implicit comparison with an imagined “rational” way to raise livestock, embedded in the promissory assemblage of the suburban in which these farmers are embedded. But the “rational” model belies the realities of working with these animals.

Farmers are keen to boost the health potential of their chickens, and antibiotics present themselves as a plausible tool for doing so. The administration of antibiotics is not a frivolous decision. Farmers agonize over their animals—they care for them deeply, and they are at the center of their lives and livelihoods. More than that, as Helen and Ahram show us, their encounters with their chickens are intimate, engaged, and about more than profit. Antibiotic use in chickens—like all medicine use—calls attention to the “inherent leakiness” of pharmaceuticals and the bodies that consume them (Hardon & Sanabria 2017). Reading farmers’ use of antibiotics as acts of irrationality means writing off how food systems, political landscapes, and colonial and capitalist modes of modernity shape and manage both humans and chickens. The narrative of irrationality works to obscure colonial histories, and delegitimize this intimate care of bodies, households, livelihoods and land. Entrepreneurial farmers like Helen must work and live amid the uncertainty and exhaustion of scaled‐up chicken farming operations. They deploy antibiotics as ways to reckon with, resuscitate, and sustain life within these haunted systems of global health.

## ENDNOTE

1 This kind of linear relationship between antibiotic use and reduced illness episodes is contested within scientific communities, and the exact causal mechanisms for understanding the relationship between improved overall health and antibiotic use in livestock are more complicated than this linearity would suggest. But farmers did observe something occurring here, and we were exposed to much conversation about this between farmers during research feedback sessions that we held with participants after the first round of research concluded.
